# Cadmium Tolerance of Perennial Ryegrass Induced by *Aspergillus aculeatus*

**DOI:** 10.3389/fmicb.2018.01579

**Published:** 2018-07-18

**Authors:** Shijuan Han, Xiaoning Li, Erick Amombo, Jinmin Fu, Yan Xie

**Affiliations:** ^1^Key Laboratory of Plant Germplasm Enhancement and Specialty Agriculture, Wuhan Botanical Garden, Chinese Academy of Sciences, Wuhan, China; ^2^Wuhan Botanical Garden, University of Chinese Academy of Sciences (UCAS), Beijing, China; ^3^The Institute for Advanced Study in Coastal Ecology, Ludong University, Yantai, China

**Keywords:** *Aspergillus aculeatus*, *Lolium perenne* L., Cd stress, physiological trait, chlorophyll *a* fluorescence transient, Cd tolerance

## Abstract

Cadmium (Cd) pollution is becoming increasingly prevalent, posing a global environmental hazard due to its negative effects on plants growth and human health. Phytoremediation is a green technology that involves uptake of Cd from the soil by a combination of plants and associated microbes. The objective of this study was to investigate the role of *Aspergillus aculeatus* in perennial ryegrass Cd tolerance. This fungus produced indole-3-acetic acid, siderophores, and 1-aminocyclopropane-1-carboxylate deaminase. Physiological traits including growth rate, turf quality and chlorophyll content were measured to evaluate the physiological responses of perennial ryegrass to Cd stress. These physiological traits were improved after inoculated with *A. aculeatus*. Inoculation of *A. aculeatus* actively reduced DTPA-Cd concentration in the soil and Cd translocation to plant shoots. Chlorophyll *a* fluorescence transient and the C/N ratio in shoots were elevated by *A. aculeatus*, which implied that the fungus could protect the photosystem II against Cd stress and increase the photosynthetic efficiency. These results suggested that *A. aculeatus* is beneficial in improving Cd tolerance of perennial ryegrass and reducing Cd-induced injuries, thus, it has promising potential for application of phytostabilization in Cd contaminated soil.

## Introduction

Cadmium (Cd) is a notorious toxic heavy metal pollutant, which is introduced into the soil mainly from the applications of P-fertilizers, atmospheric deposition, sewage sludge, and smelting of metals (Smolders and Mertens, 2013). Cd with high mobility can be assimilated by plants and then translocated into edible parts and eventually enter the food chain to undermines animals and human health (Wagner, 1993). Moreover, increased accumulation of Cd in human body can lead to bone disease (van de Mortel et al., 2008). Hence, finding an effective remediation for Cd-contaminated environments is an ecological priority.

Phytoremediation is a relatively new technique, utilizing plants to remediate polluted soils (Glick, 2003). Categorically, it has been classified into several approaches such as phytoextraction, phytostabilization, and phytostimulation. However, there are some limiting factors to this technology. For example, there need for a longer treatment time to reach a satisfying effect because the plants with poor adaptability grow slowly and absorb subtle amounts of heavy metal from the contaminated soil (Cunningham et al., 1995).

Many microbial communities are sensitive to heavy metals. It has been reported that various soil microorganisms can dispose toxic chemical compounds in the soil by degradation, hence such microbes are commonly applied in bioremediation (Hallberg and Johnson, 2005; Kao et al., 2006; Umrania, 2006). Bioremediation approaches based on microbial-plant combinations might contribute to the success of phytoremediation and mitigating the heavy metal-polluted soil.

Plant growth-promoting microbes have attracted remarkable attention because they can greatly accelerate the plants bioremediation process by stimulating growth through various mechanisms. *Aspergillus aculeatus* (*A. aculeatus*), which was isolated from a Cd contaminated site, showed tolerance to Cd and was identified as a kind of Cd-resistant fungus strain (Xie et al., 2014b). Previous research has also confirmed that *A. aculeatus* inoculation on bermudagrass could improve turf quality and assuage the Cd toxicity for plants grown in Cd polluted soil (Xie et al., 2014b). Furthermore, this fungus has the characteristics of absorbing Cd and therefore has been widely used to remove Cd from industrial wastewater (Pandey and Banerjee, 2012). Our preliminary study has shown that the fungus enters roots outer epidermic cells of ryegrass grown in *A. aculeatus*-inoculated substrate (data not shown). Consequently, the fungus has become a suitable plant growth-promoting microbe in Cd-polluted soil and has a high potential to improve the efficiency of phytoremediation.

Perennial ryegrass (*Lolium perenne* L.) is an important grass which is widely used in many countries. It can enrich Cd in the soil (Xiong et al., 2007; Luo et al., 2011). Previous investigation has indicated that perennial ryegrass can grow normally on Cd-polluted soil which is attributed to its higher tolerance to Cd (Arienzo et al., 2004). Therefore, the perennial ryegrass with high biomass yields is an appropriate species to apply in the remediation of heavy metals contaminated soil.

The effects of *A. aculeatus* on turf quality, chlorophyll content and photochemical efficiency on bermudagrass exposed to Cd stress have been well addressed, but little is known about the influence on perennial ryegrass in response to Cd stress. Thus, for illuminating the potential function of *A. aculeatus* in phytoremediation, it is necessary to study on the physiological traits of perennial ryegrass exposed to Cd stress after inoculated with this fungus. The toxicity of Cd on plants can be evaluated by measuring some associated physiological traits of plants under the Cd stress. It has been confirmed that Cd can tamper with many biochemical and physiological processes in plants such as photosynthesis, respiration and nitrogen metabolism (Gupta et al., 2009; Zhang et al., 2009; Piotrowska et al., 2010). In addition, Cd accumulation in plants causes stunted plant growth, chlorosis in plant tissue, lower uptake of water and nutrients by roots and higher electrolyte leakage, which could be assessed as membrane damage by measuring the ion leakage of the cell (Garg and Aggarwal, 2012).

In this study, we designed a pot experiment to measure these physiological traits including the growth rate, chlorophyll content, chlorophyll *a* fluorescence transient, water content, electrolyte leakage, some critical ions content and C/N ratio in the plants. The study was designed to investigate perennial ryegrass physiological responses to Cd stress after inoculation with *A. aculeatus* and what influence the *A. aculeatus* has on the capacity of perennial ryegrass to resist Cd stress.

## Materials and methods

### Preparation of growth matrix

*A. aculeatus* preserved in Martin medium at the −80°C was activated by streaking the culture fresh Martin medium, and incubation for 48 h at 30°C. After a 48 h of propagation phase, the *A. aculeatus* were transferred into flasks containing 150 mL of liquid Martin medium (Xie et al., 2014b). All flasks inoculated with *A. aculeatus* were placed in the Orbital Shaker Incubator at 180 rpm cultured for 72 h at 30°C.

The substrate was made by mixing sand with sawdust and stirring thoroughly at a ratio of 2:1 (v/v) then the mixture was divided into 30 groups, of 500 g each. All substrates were sterilized before use and half of them were inoculated with the same amount of *A. aculeatus* which was filtered with multilayer gauze from the liquid medium after 72 h of establishment and washed with the sterile water in advance. Then all the substrates were cultured at 30°C for 48 h before moved into container (15 cm in diameter and 20 cm tall). The substrate prepared previous were considered as the “soil” in the following context.

### Plant materials and growth conditions

Seeds of the perennial ryegrass (cv. ‘lark’) were selected based on their uniformity and sown in plastic pots (13 cm in diameter and 15 cm deep) filled with solid growth substances (nutrient soils: sand = 2:1, v/v). All the pots were maintained in a plant growth chamber for 45 days with a daily temperature regime of 26/20°C (day/night), 14 h daylight, photosynthetic active radiation (PAR) levels of 720 μmol m^−2^s^−1^ and irrigated with 200 mL half-strength Hoagland's solution (Hogland and Arnon, 1950) every week.

After a 45-days period of growth, the roots were taken out from the pots and rinsed with tap water until all the growth substances adhered to the root surface were removed. An equal proportion of plants with similar growth vigor were selected and transplanted into the soil above-mentioned. Before the Cd treatments, all the plants were placed in plant growth chamber for 2 weeks with the same conditions as before.

### Experimental treatments

After 2-weeks of adaptation period, perennial ryegrass was subjected to three Cd concentration treatments: 0 (half-strength Hoagland nutrition as the control), 200 and 400 mg kg^−1^ Cd (CdSO_4_·8/3 H_2_O dissolved in the half-strength Hoagland nutrition). The 30 pots containing grass were distributed into 6 groups in a randomized complete block design and were used to perform different treatments, including control (0 Cd, 0 Cd + *A. aculeatus*), Cd treatment only (200 Cd, 400 Cd), Cd treatment coupled with *A. aculeatus* (200 Cd+*A. aculeatus*, 400 Cd+*A. aculeatus*). The plants were trimmed to equal height (10 cm tall) in order to maintain consistency. The fungus-inoculated pots were supplemented with 15 mL of fungal suspension at the rhizosphere of plants before the Cd treatment.

The final Cd concentration was reached by irrigating soil with 100 mL of half-strength Hoagland solution containing set volume of 10 mM CdSO_4_ on the first, 5th, 10th, and 15th day after the treatment was initiated. On the other hand, the control groups were also irrigated with the same volume of half-strength Hoagland solution without CdSO_4_. There was a saucer under each pot to avoid fungus leaching and make sure all of the irrigation solution was reabsorbed. The whole treatment process was conducted in plant growth chamber with the above-mentioned conditions and lasted for 18 days.

### Measurements

#### IAA, siderophore and ACC deaminase production

The iodole-3-acetic acid (IAA) concentration in culture was detected with the presence of L-tryptophan according to the procedures mentioned in previous study (Gordon and Weber, 1951). The absorbance values of pink color formed after 25 min incubation at 530 nm were read and calibrated with calibration curve of pure IAA to calculate the IAA concentration in culture. The siderophore secreted by the fungus was detected following the procedure in previous study (Schwyn and Neilands, 1987) with the supplement of dye Chrome azurol S (CAS) in blue agar plates. The siderophore excretion generated by the fungus developed into orange halos around the colonies on blue agar. Otherwise, the amount of α-ketobutyrate (α KB) generated by the enzymatic hydrolysis of 1-aminocyclopropane-1-carboxylate (ACC) were monitored to determine the ACC deaminase activity (Saleh and Glick, 2001).

#### Relative growth rate

Relative growth rate (RGR) was calculated by Equation (1). The RGR of the shoot was calculated by measuring the vertical average height of grass recorded as H_0_ before Cd treatment; after the treatment the height recorded as H_t_. The interval time between two measurements were recorded as Δt. In order to determine the root RGR, the final average root length was measured when the roots harvested, the initial length of root was set at 7 cm.

(1) $$\begin{array}{r} RGR=\left(H_{t}-H_{0}\right)/\Delta t \end{array}$$

#### Turf quality

Turf quality (TQ) was evaluated visually by three different researchers and was based on the degree of shoot wilting and leaf senescence, turf grass color and plant density using a regime of nine points where 9 means the grass is uniform, dense and green; 0 represents senesced, yellow and dead grass; 6 is minimum acceptable level of quality (Turgeon, 2002).

#### Chlorophyll content

Chlorophyll (Chl) content was measured according to the previous method (Hiscox and Israelstam, 1979) and the 3rd fully expanded blades of the grass were collected and cut into pieces.

#### The fluorescence transient of Chlorophyll

Chlorophyll *a* fluorescence transient (OJIP transient) were measured using a pulse-amplitude modulation fluorometer (PAM 2500, Heinz Walz GmbH. Effeltrich, Germany), and the time resolution was set at 10 μs. The 3rd fully expanded leaves of the ryegrass were selected for subsequent measurement of fluorescence transient with 8 replicates of each treatment. Before the measurement, a dark adaptation of leaves was conducted for 25 min. Chlorophyll *a* fluorescence transient containing many fluorescence parameters and crucial information about photosynthesis can be calculated by the JIP-Test according to previous studies (Han et al., 2009; Chen et al., 2013).

#### The shoot water content

At the end of the assay, samples were separated into two parts: shoots and roots. The shoots of ryegrass were weighted and recorded as the fresh weight (FW). Subsequently, the shoots were oven killed for 30 min at 105°C, and then dried for 3 days at 80°C until a constant weight was attained. The samples were recorded as the dry weight (DW). Finally, the shoot water content was calculated by the Equation (2):

(2) $$\begin{array}{r} SWC\left(\%\right)=\left(\text{FW}-\text{DW}\right)\times 100/\text{FW} \end{array}$$

#### The electrolyte leakage

The 3rd fully expanded leaves of the ryegrass per pot were collected to determine the electrolyte leakage (EL). The samples were washed several times using deionized water and then dried out with filter paper. After washing, the leaves were cut into small pieces and 0.2 g was weighed and added into 50-mL tubes containing 10 mL deionized water. The tubes were shaken on the table concentrator at 25°C for 24 h then the first electric conductivity was measured using the conductivity meter (JENCO-3173, JENCO Instruments, Inc., San Diego, CA, USA) and recorded as A. Subsequently, the tubes were autoclaved at 100°C for 15 min. When the tubes were cooled to room temperature and the second electric conductivity value was determined recorded as B. The EL of leaves was calculated by the Equation (3):

(3) $$\begin{array}{r} \text{EL}\left(\%\right)=A/B\times 100 \end{array}$$

#### Ion concentration and soil pH

The shoots of perennial ryegrass were harvested individually after 18-days of treatments and then washed using sterilized ultra-purified water for three times. The leaves were dried for 3 days at 80°C and then ground into fine powder using multichannel tissue ball milling instrument (Scientz-192, Scientz biotechnology, GmbH, Ning Bo, China). The powder of shoot was weighed (0.1 g) separately and digested with a mixture of concentrated HNO_3_, HCl and HF (5:2:2, v/v/v). After digesting, the mixture was placed in heating plate at 135°C for 30 min to eliminate all acids. The residual solution was transferred into a 50-mL volumetric flask and rinsed with 1% HNO_3_ solution to 50 mL and evenly mixed. The purified solution was filtered through a 0.45 μm-millipore membrane and collected into a 15-mL tubes which was used to determine the Cd concentration by ICP-MS (X Series 2, Perkin Elmer, USA).

The soil DTPA-Cd concentrations extracted by diethylene-triaminepentaacetic acid (DTPA) and the other ions concentration (P, Cu, Ca, Mg, and Mn) were determined using ICP-OES (OPTIMA 8000DV, Perkin Elmer, USA). Each treatment had five replicates. The soil extracted by 10 mM CaCl_2_ solution was utilized for determining the pH of the soil under different treatments.

#### The content of total organic C and N

The total organic C (TOC) and total N (NC) concentration in shoots and roots were measured by a stable isotope mass spectrometer (Delta V Advantage, Thermo Finnigan, Germany), running in continuous flow mode. Subsamples were oven-dried and grounded into fine powder of shoot samples (0.3–0.4 mg), root samples (0.5–0.55 mg), and urea (0.3–0.4 mg) and regarded as reference materials. The materials were put into tin capsules separately and sealed, and then placed into different holes of the automatic sampler in proper order before analysis. Finally, the content of TOC, TN, and the C/N ratio in shoot and root were determined by this apparatus.

### Statistical analyses

Each treatment had five replicates and all the values of this experiment were expressed as mean ± SD (standard deviation). The significant difference of means was determined based on Student-Newman-Keuls test (SNK) at 5% probability level. The data were analyzed by one-way analysis of variance (One-Way ANOVA) using SPSS statistical software package (version 20.0; SPSS Inc., Chicago, IL, USA).The graphs were created using SigmaPlot 12.3 (Systat Software, Richmond, CA) and Origin 9.0 (Origin Lab Inc., Hampton, USA) for OJIP transient.

## Results

### Characteristics of the strains

As showed in Table 1, the *A. aculeatus* strains were capable of producing siderophore, IAA with the presence of L-tryptophan in the medium and enhancing the ACC deaminase activity in culture. Furthermore, the Siderophore production and ACC deaminase activity significantly decreased with the Cd concentration increase, whereas the concentration of IAA was slightly decreased under Cd stress.

**Table 1:** Characteristics of the *A. aculeatus* strains.

	**Cd concentration (mg L^−1^)**				
	**0**	**5**	**10**	**20**	**50**
**Indole-3-acetic acid (pmol L^−1^)**	11.30a	8.85a	9.28a	9.05a	10.68a
**Siderophore production (%)**	26.15ab	29.25a	24.90ab	22.20bc	18.40c
**ACC deaminase activity (μM α KB mg^−1^ h^−1^)**	26.30a	14.10b	8.20c	4.88d	4.18d

*Values are means of five samples. Rows marked with the same lower case letter were not significantly different among treatments based on a SNK test at P < 0.05*.

### *A. aculeatus* improved the growth of ryegrass under Cd stress

**Figure 1:**
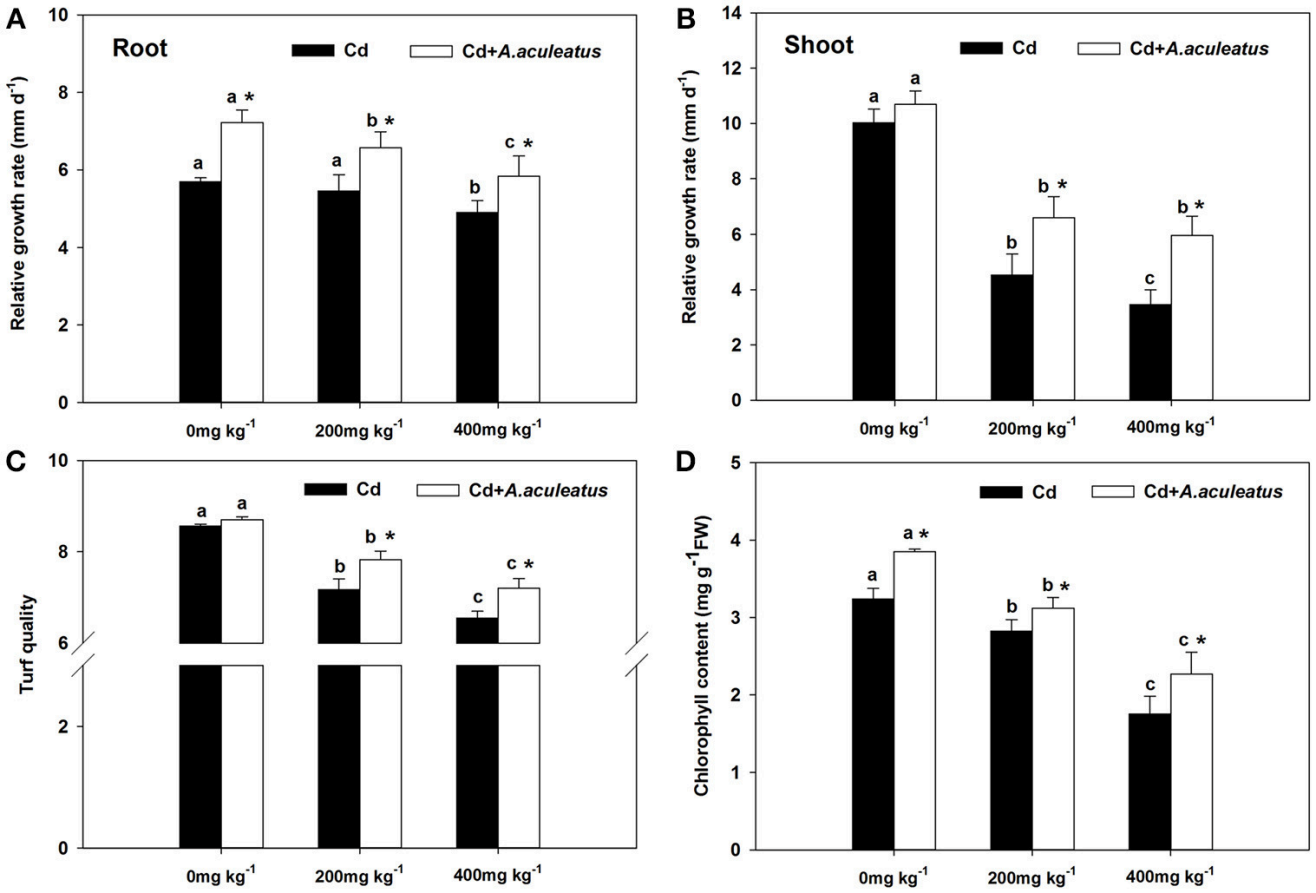
Effects of *A. aculeatus* on the relative growth rate in the root **(A)** or shoot **(B)** and the turf quality **(C)**, chlorophyll content **(D)** of ryegrass grown with or without *A. aculeatus* under 0, 200 and 400 mg kg^−1^ Cd stress. Error bars, SD. Bars marked with same lower-case letter for a given treatment (i.e., *A. aculeatus*-inoculated or non-inoculated) were not significantly different for the comparison of Cd concentrations based on a SNK test at *P* < 0.05. Columns labeled with the ^*^ represent significant difference for the comparison between different inoculation treatments at the same level of Cd stress at *P* < 0.05 (SNK test).

The non-inoculated plants showed stunted growth compared with inoculated under the Cd treatment. The relative growth rate of shoots and roots were both decreased when subjected to 200 mg kg^−1^ and 400 mg kg^−1^ Cd compared to the plants without Cd stress, to a sharper decline for the shoots between the plants without Cd stress and Cd-treated (Figures 1A,B). Moreover, the results indicated that the *A. aculeatus* could also cause a greater increase in root RGR under Cd stress (Figure 1A). Moreover, the RGR of the ryegrass shoots or roots inoculated with *A. aculeatus* was higher than those non-inoculated plants under the same level of Cd treatment.

Cd stress had the negative effect on turf quality and chlorophyll content which were both declined gradually with the increase of Cd (Figures 1C,D). Furthermore, *A. aculeatus*-inoculated plants had a greater level of turf quality and chlorophyll content than non-inoculated plants regardless of Cd concentrations. In addition, there were significant differences in chlorophyll content between the inoculated and non-inoculated plants (*P* < 0.05).

### *A. aculeatus* reduces electrolyte leakage in Cd-treated ryegrass

**Figure 2:**
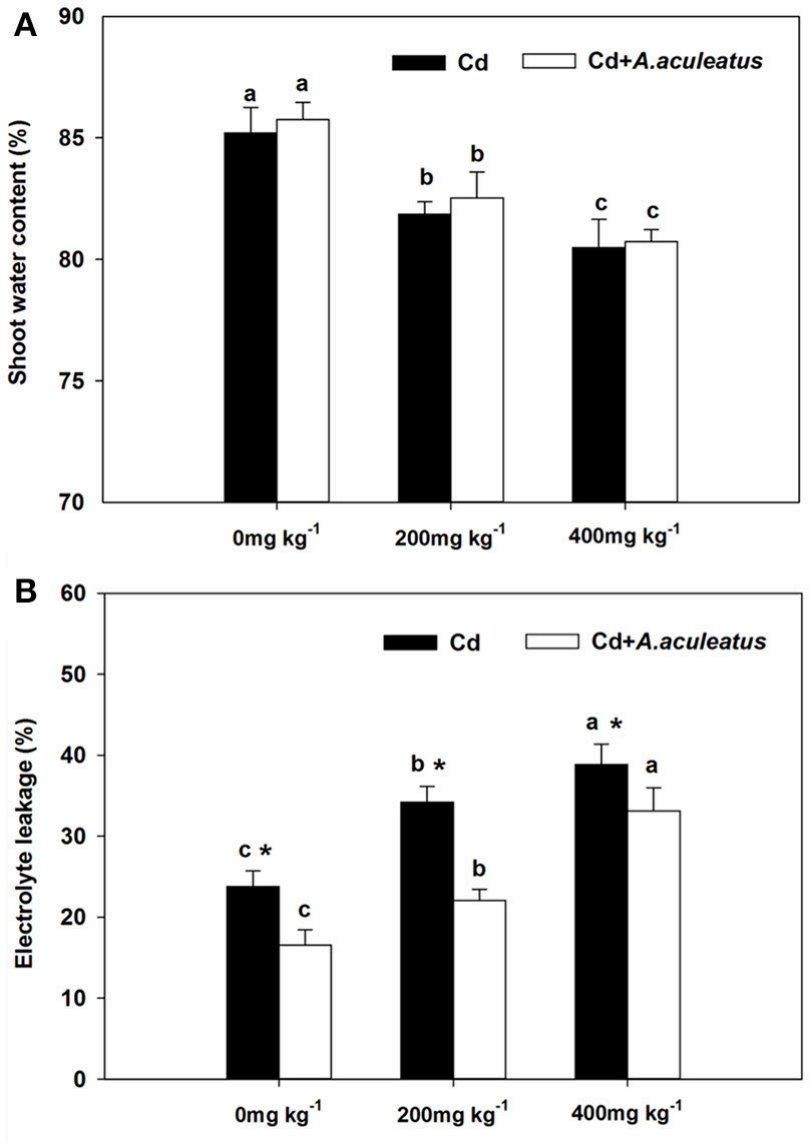
Effects of *A. aculeatus* on the water content of the shoot **(A)** and the electrolyte leakage **(B)** for perennial ryegrass subjected to different levels of Cd concentrations. Error bars, SD. Bars marked with same lower-case letter for a given treatment (i.e., *A. aculeatus*-inoculated or non-inoculated) were not significantly different for the comparison of Cd concentrations based on a SNK test at *P* < 0.05. Columns labeled with the ^*^ represent significant difference for the comparison between different inoculation treatments at the same level of Cd stress at *P* < 0.05 (SNK test).

The water content of shoot and the electrolyte leakage of leaf responded differently to increasing Cd concentration (Figure 2). The former declined but the latter was improved by Cd stress throughout the assay. Although there was no obvious difference in water content between the inoculated and non-inoculated plants, the water content of *A. aculeatus*-inoculated regimes was slightly higher than non-inoculated plants under the same Cd exposure (Figure 2A). The electrolyte leakage of leaf samples was greatly enhanced with the rising Cd concentration (Figure 2B).The electrolyte leakage in leaves of inoculated plants was markedly decreased in contrast with non-inoculated plants when subjected to same Cd stress.

### *A. aculeatus* enhances photosynthetic efficiency of Cd-treated ryegrass

**Figure 3:**
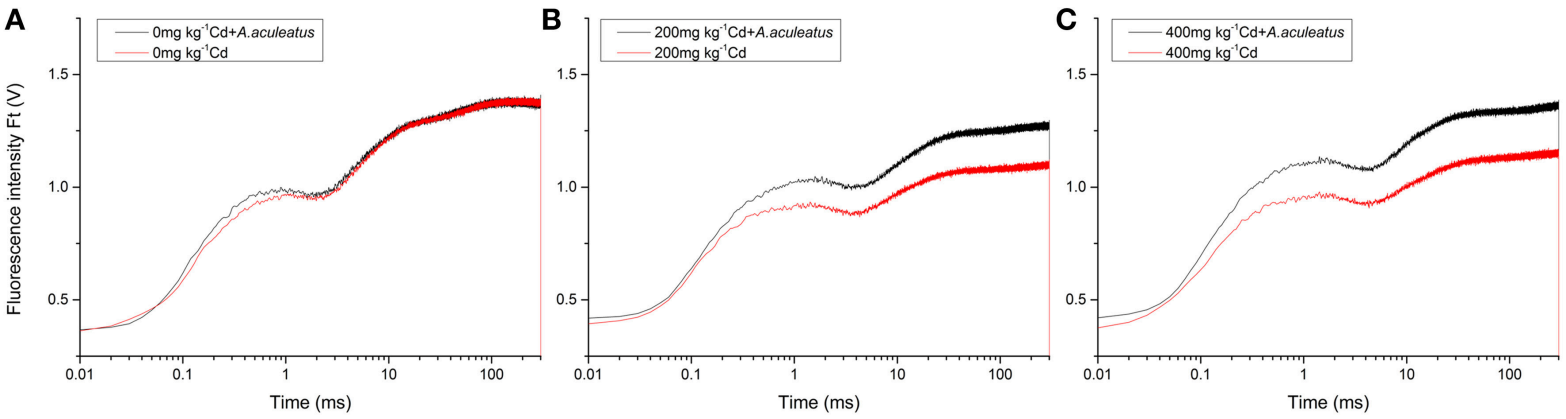
Effects of *A. aculeatus* on OJIP fluorescence transient for perennial ryegrass subjected to different levels of Cd concentrations. Plants were treated with 0 **(A)**, 200 **(B)** and 400 mg kg^−1^ **(C)** Cd treatments.

OJIP fluorescence transient curves of the plants under different treatments were measured and plotted in Figure 3. There were different responses in OJIP fluorescence transient of ryegrass under Cd stress in the presence and absence of *A. aculeatus*. No significant difference between *A. aculeatus*-inoculated and non-inoculated plants was found in the condition without Cd stress (Figure 3A). However, when subjected to 200 mg kg^−1^ Cd stress, the plants without inoculation dropped dramatically compared to the inoculated plants (Figure 3B). Furthermore, the results showed that the ryegrass inoculated with *A. aculeateus* appeared a higher OJIP fluorescence transient than non-inoculated ryegrass under 200 and 400 mg kg^−1^ Cd stress (Figures 3B,C).

The OJIP transient curve of the inoculated plants were not significantly affected by Cd stress compared with the plants without Cd treatment. Consequently, the OJIP transients of ryegrass leaves were significantly improved by *A. aculeatus* under Cd stress. Interestingly, both of the *A. aculeatus*-inoculated and non-inoculated plants had a slight increase in the OJIP transient curve when exposed to 400 mg kg^−1^ Cd treatment than 200 mg kg^−1^ Cd treatment respectively. Moreover, the J-step and I-step in OJIP fluorescence transient of plants under Cd stress were postponed contrasted with the control. The Cd stress significantly decreased OJIP fluorescence transient of ryegrass leaves, to a greater decline in the non-inoculated plants. In order to further explore the function of *A. aculeatus* on PSII in ryegrass under Cd stress, the JIP-test was analyzed based on the OJIP transient curves and the fluorescence parameters were listed in Table 2.

**Table 2:** Photosynthetic parameters calculated by analyzing the JIP-test of OJIP fluorescence transients.

	**CK+A**	**CK**	**200Cd+A**	**200Cd**	**400Cd+A**	**400Cd**	**Definitions**
**DATA EXTRACTED FROM THE RECORDED OJIP FLUORESCENCE TRANSIENT CURVES**							
F_0_ = 20μs	0.36b	0.38a	0.44a^*^	0.36a	0.42a^*^	0.37a	Fluorescence at time t after onset of actinic illumination
F_K_	0.84b^*^	0.91a	0.93a^*^	0.81b	0.96a^*^	0.82b	Fluorescence value at 300 μs
F_J_	0.97b	0.97a	1.04a^*^	0.89a	1.10a^*^	0.90a	Fluorescence value at the J-step of OJIP
F_I_	1.30a	1.27a	1.23a^*^	1.03b	1.28a^*^	1.00b	Fluorescence value at the I-step of OJIP
F_P_ = F_M_	1.43a	1.38a	1.26b^*^	1.08b	1.43a^*^	1.05b	Fluorescence value at the peak of OJIP test
V_J_	0.57b	0.59c	0.47c^*^	0.74b	0.67a^*^	0.78a	Relative variable fluorescence at the J-step
V_I_	0.88a	0.89a	0.61b^*^	0.94a	0.85a	0.93a	Relative variable fluorescence at the I-step
M_o_	1.80b^*^	2.11b	1.53c^*^	2.49a	2.14a^*^	2.66a	Approximate value of the initial slope of fluorescence transient curves
**QUANTUM YIELDS AND EFFICIENCIES/PROBABILITIES**							
ϕP0	0.75a	0.73a	0.65c	0.67b	0.71b^*^	0.64b	Maximum quantum yield for primary photochemistry, namely F_V_ /F_M_
ϕE_0_	0.32a	0.30a	0.18c	0.17b	0.23b^*^	0.15b	Quantum yield of the electron transport flux from Q_A_ to Q_B_
ϕR_0_	0.09a	0.08a	0.03b	0.04a	0.10a	0.04a	Quantum yield for reduction of end electron acceptors at the PSI acceptor side
ΨE_0_	0.43b	0.41a	0.53a^*^	0.26b	0.33c^*^	0.22c	Efficiency/probability with which a PSII trapped electron is transferred from Q_A_ to Q_B_
δR_0_	0.29b	0.26a	0.73a^*^	0.24a	0.43b	0.31a	Efficiency/probability with which an electron from Q_B_ is transferred until PSI acceptors
**SPECIFIC ENERGY FLUXES (PER ACTIVE PSII REACTION CENTER)**							
ABS/RC	0.76b^*^	0.90b	0.72b^*^	1.22a	1.02a^*^	1.33a	Absorbed photon flux per RC
γRC2	0.19a^*^	0.17a	0.17b	0.17a	0.18a^*^	0.16a	Probability that a PSII Chl molecule functions as RC
RC/ABS	1.31a^*^	1.12a	1.41a^*^	0.83b	1.00b^*^	0.76b	Number of Q_A_ reducing RCs per PSII antenna Chl
TR_0_/RC	3.19a^*^	3.59a	3.29a	3.37a	3.20a	3.43a	Trapped excitation flux (leading to Q_A_ reduction) per RC
ET_0_/RC	1.39b	1.48a	1.76a^*^	0.88b	1.05c^*^	0.77c	Electron transport flux (further than ${\text{Q}}_{\text{A}}^{-}$) per RC
RE_0_/RC	0.40b	0.38a	1.28a^*^	0.21a	0.48b	0.23a	Electron flux reducing end electron acceptors at the PSI acceptor side, per RC
**PERFORMANCE INDEXES (PI, COMBINATION OF PARAMETERS)**							
PI_ABS_	0.53a^*^	0.38a	0.43b^*^	0.14b	0.26c^*^	0.10b	PI (potential) for energy conservation from exciton to the reduction of intersystem electron
PI_total_	0.22b	0.14a	1.13a^*^	0.06b	0.16b^*^	0.05b	PI (potential) for energy conservation from exciton to the reduction of PSI end acceptors

The mean values of five repetitions listed in a column followed by same letters for the comparison among the different levels of Cd stress at the same inoculation treatments (i.e., A. aculeatus-inoculated or non-inoculated) were not significantly different based on a SNK test at P < 0.05. Means followed by the

* *represent significant difference for the comparison between different inoculation treatments at the same level of Cd stress at P < 0.05 (SNK test). 200Cd+A, inoculated plants treated with 200 mg kg^−1^ Cd stress; 200Cd, only treated with 200 mg kg^−1^ Cd stress; 400Cd+A, inoculated plants treated with 400 mg kg^−1^ Cd stress; 400Cd, only treated with 400 mg kg^−1^ Cd stress*.

The basic fluorescence parameters values extracted directly from the OJIP fluorescence transient curves displayed notable differences after treatments, such as F_0_, F_K_, F_J_, F_I_, and F_P_ = F_M._. Five basic fluorescence parameters of inoculated ryegrass were significantly higher than the non-inoculated ryegrass when subjected to the same level of Cd stress (Table 2). Moreover, the plants treated with 400 mg kg^−1^ Cd and inoculated with *A. aculeatus* had a set of higher basic fluorescence parameters compared to the control groups, except for F_I_ and F_P_. The inoculated plants under 200 mg kg^−1^ Cd treatment had higher F_0_, F_K_, F_J_ but a lower F_P_ than control groups, overall, there was no significant difference in F_I_. The treatment with Cd stress only resulted in a significant increase in V_J_, V_I_, and M_0_ which were calculated based on basic fluorescence parameters, however, after inoculation, the parameters were decreased remarkably than those Cd only treated.

The Cd stress significantly declined the ϕP_0_, ϕE_0_, ϕR_0_, and ΨE_0_ values which reflected Quantum yields and efficiencies. Furthermore, after inoculation with *A. aculeatus*, some of the parameters such as ϕE_0_, ΨE_0_, and δR_0_ were significantly improved caused by *A. aculeatus*. Particularly under the 400Cd+A treatment, ϕP_0_, ϕR_0_, and ΨE_0_ were also enhanced compared to the non-inoculated at the same levels of Cd treatment. The parameters of specific energy fluxes which were referred to ABS/RC, γRC2, RC/ABS, TR_0_/RC, RE_0_/RC, and ET_0_/RC also changed remarkably under different treatments. There was a greater decline in RC/ABS, ET_0_/RC and RE_0_/RC which were induced by Cd stress. Meanwhile, a lager rise in the ABS/RC of the leaves was observed after the Cd stress compared to the control, especially under the Cd only treatment regime. Inoculation with *A. aculeatus* significantly enhanced the parameters of specific energy fluxes except for the ABS/RC and TR_0_/RC compared to non-inoculated plants under the same levels of Cd stress. Both of the PI_ABS_ and PI_total_ were crucial indices, which play important roles in describing the overall activity of PSII. As shown in Table 2, there was a greater decline in both the PI_ABS_ and PI_total_ after Cd exposure. However, inoculation with *A. aculeatus* could significantly improve the values of the two indices. Interestingly, inoculated plants even under the Cd stress also had a higher PI_total_ values than control groups without Cd treatment.

### Changes in Cd uptake and ionic homeostasis

**Figure 4:**
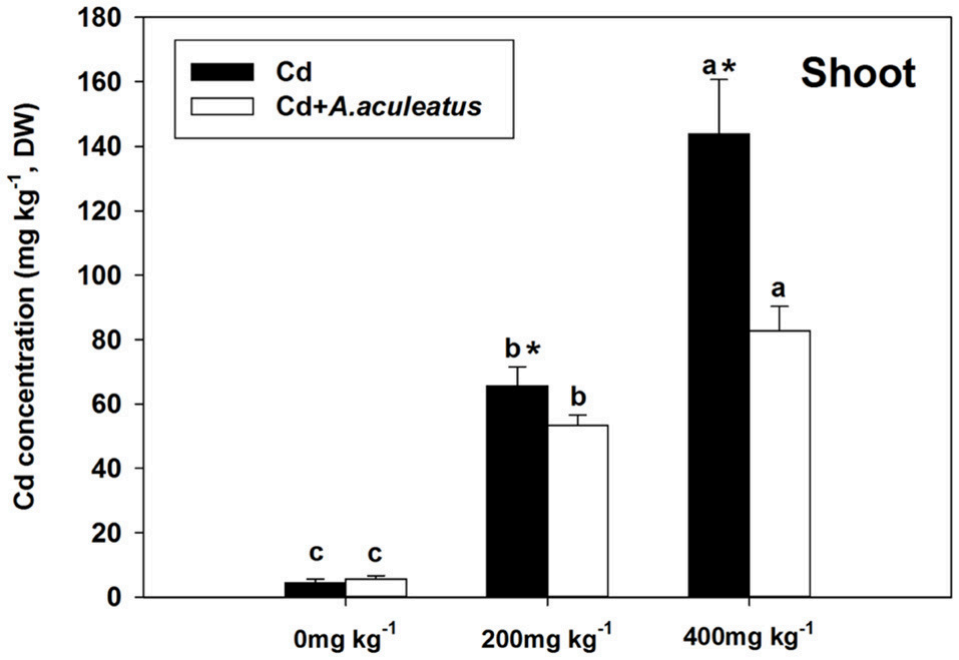
Effects of *A. aculeatus* on plant Cd concentration accumulated in shoot for perennial ryegrass subjected to different levels of Cd concentrations. Error bars, SD. Bars marked with same lower-case letter for a given treatment (i.e., *A. aculeatus*-inoculated or non-inoculated) were not significantly different for the comparison of Cd concentrations based on a SNK test at *P* < 0.05. Columns labeled with the ^*^ represent significant difference for the comparison between different inoculation treatments at the same level of Cd stress at *P* < 0.05 (SNK test).

Cd concentration in the plants was strongly improved compared with the plants without Cd treatment after the 18-days Cd stress, to a larger extent in the non-inoculated plants. The significant decline of Cd concentration in shoot were observed in inoculated plants when exposed to 200 and 400 mg kg^−1^ Cd stress (Figure 4).

Different ions contents in shoot and root tissues responded differently to Cd stress (Table 3). Ca^2+^ concentrations in shoot declined profoundly with the increasing Cd levels, however, the results shown in root were adverse. There was no difference in Ca^2+^ concentration between *A. aculeatus*-inoculated plants and non-inoculated regime shoots. *A. aculeatus* inoculation significantly improved the Ca^2+^ concentrations in root compared with the Cd only treated under the same Cd stress. The variation tendency of Cu^2+^ concentrations in the plant was similar with the Ca^2+^. Cd had no significant influence on Cu^2+^ concentrations in shoot but *A. aculeatus* improved the Cu^2+^ concentrations in root notably after exposed to 400 mg kg^−1^ Cd stress. When plants exposed to 400 mg kg^−1^ Cd stress, Mg^2+^ in shoot was significantly improved but in root were decreased pronouncedly. Inoculation with *A. aculeatus* caused a slight increase of Mg^2+^ in root when plants subjected to the 400 mg kg^−1^ Cd stress. Furthermore, a significant increase in Mg^2+^ content in inoculated root was observed compared to non-inoculated counterpart when subjected to 200 mg kg^−1^ Cd stress.

**Table 3:** Effects of A. aculeatus on calcium (Ca), cuprum (Cu), magnesium (Mg), manganese (Mn), and phosphorus (P) concentrations of perennial ryegrass subjected to different levels of Cd concentrations.

**Cd treatment (mg kg^−1^)**	***A. aculeatus***	**Root**					**Shoot**				
		**Ca^2+^ (mg g^−1^DW)**	**Cu^2+^ (mg g^−1^DW)**	**Mg^2+^ (mg g^−1^DW)**	**Mn (mg g^−1^DW)**	**P (mg g^−1^DW)**	**Ca^2+^ (mg g^−1^DW)**	**Cu^2+^ (mg g^−1^DW)**	**Mg^2+^ (mg g^−1^DW)**	**Mn (mg g^−1^DW)**	**P (mg g^−1^DW)**
0	+	6.70b^*^	0.14b	2.31a	0.38b^*^	3.29*a*b	7.34a	0.12*a*	0.78c^*^	0.16a	5.21a^*^
	–	2.59b	0.14b	2.17a	0.27a	3.19a	7.35a	0.13a	1.89b	0.14a	4.05a
200	+	7.40b^*^	0.15b	2.13b^*^	0.31c^*^	2.90b^*^	6.03b	0.11b	1.76b	0.11c	3.50b
	–	5.58a	0.13b	1.95b	0.20b	2.42b	6.34b	0.10b	1.96b	0.10b	3.72a
400	+	10.90a^*^	0.17a^*^	1.87b	0.57a^*^	3.53a^*^	6.76*a*b	0.11b	2.64*a*	0.13b^*^	4.90a^*^
	–	6.00a	0.15a	1.59c	0.28a	2.72b	6.21b	0.11b	2.61*a*	0.11b	3.76a

The mean values of five repetitions listed in a column followed by same letters for the comparison among the different levels of Cd stress at the same inoculation treatments (i.e., A. aculeatus-inoculated or non-inoculated) were not significantly different based on a SNK test at P < 0.05. Means followed by the

* *represent significant difference for the comparison between different inoculation treatments at the same level of Cd stress at P < 0.05 (SNK test)*.

On the other hand, the accumulation of Mn in shoot and root were all reduced after 200 mg kg^−1^ Cd treatment, in contrast to the control groups without Cd treatment. Interestingly, when plants exposed to 400 mg kg^−1^ of Cd stress, there was a slight rise in Mn concentration both in shoots and roots compared to 200 mg kg^−1^ Cd level. By contrast, inoculation of *A. aculeatus* significantly increased the content of Mn in root (*P* < 0.05). The P concentrations in the plant displayed similar tendency with Mn. In shoot, the P concentrations were dramatically improved by *A. aculeatus* except at 200 mg kg^−1^ Cd exposure. Furthermore, *A. aculeatus* resulted in a significant increase in P concentration in root contrasted with uninfected plants under the same Cd treatment.

### Changes in TOC and TN

The shoot TN content declined and showed a notable difference with the increasing Cd concentration (P < 0.05) from the Table 4. In addition, the ryegrass inoculated with A. aculeatus had a higher level of TN in shoot in contrast to uninfected plants when subjected to the same Cd stress. However, in root, TN content was gradually enhanced under different Cd stress, and there was no significant difference between infected and uninfected plants. TOC content in the shoot was not affected significantly by Cd treatment, but a notable decline was observed in root after the Cd stress. The results showed that A. aculeatus had no evident influence on the content of TOC compared to uninfected plants.

**Table 4:** Effects of *A. aculeatus* on the content of total organic C and total N in perennial ryegrass subjected to different levels of Cd concentrations.

**Cd treatment (mg kg^−1^)**	**A. aculeatus**	**Root**			**Shoot**		
		**TN (%)**	**TOC (%)**	**C/N ratio (%)**	**TN (%)**	**TOC (%)**	**C/N ratio (%)**
**0**	+	1.59b	39.82a	25.19a	4.09a^*^	40.66a	9.97b^*^
	**–**	1.58b	39.14a	25.54a	3.22a	40.35a	12.64c
**200**	+	1.52b^*^	38.19b	25.32a^*^	3.16b^*^	39.62b	12.57a^*^
	**–**	1.69ab	37.88b	22.50ab	2.84b	40.06a	14.14b
**400**	+	1.78a	37.23c	20.93b	3.13c^*^	40.83a^*^	13.13a^*^
	**–**	1.81a	37.35b	20.81b	2.53c	39.31a	15.55a

The mean values of five repetitions listed in a column followed by same letters for the comparison among the different levels of Cd stress at the same inoculation treatments (i.e., A. aculeatus-inoculated or non-inoculated) were not significantly different based on a SNK test at P < 0.05. Means followed by the

* *represent significant difference for the comparison between different inoculation treatments at the same level of Cd stress at P < 0.05 (SNK test)*.

The C/N ratio in the shoot were markedly increased after Cd treatment (P < 0.05). Furthermore, the ratio of C/N in the shoot was decreased remarkably by inoculation with A. aculeatus compared to uninfected plants. Nevertheless, in root, C/N ratio was declined significantly with the increasing levels of Cd stress. The ratio of C/N in root under the same Cd treatment was not sensitive to A. aculeatus, and no significant difference was observed in C/N ratio between inoculated and non-inoculated plants in root.

### DTPA-Cd concentration and pH value in the soil affected by A. aculeatus

**Figure 5:**
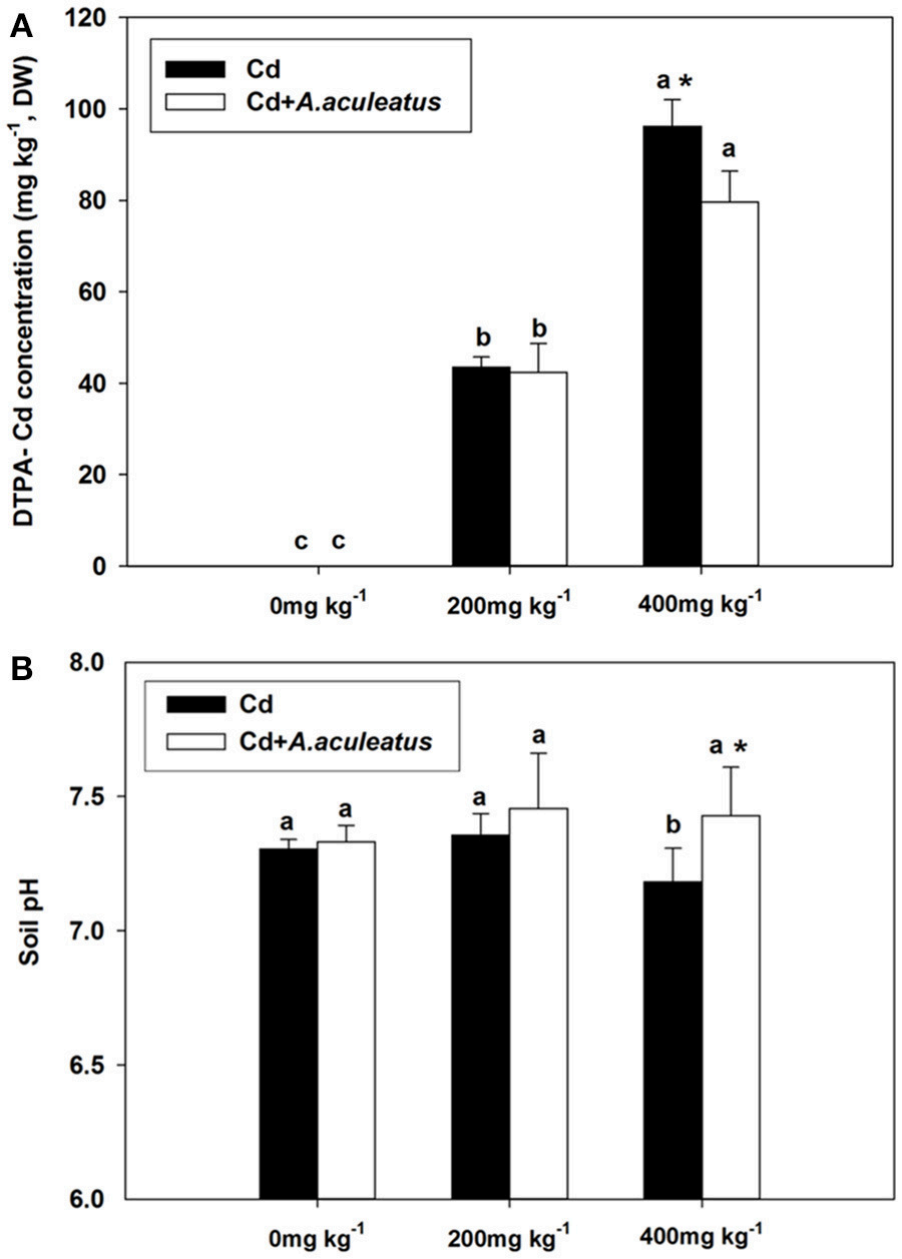
Effects of *A. aculeatus* on the DTPA-Cd concentration **(A)** and pH **(B)** in the soil for perennial ryegrass subjected to different levels of Cd concentrations. Error bars, SD. Bars marked with same lower-case letter for a given treatment (i.e.,*A. aculeatus*-inoculated or non-inoculated) were not significantly different for the comparison of Cd concentrations based on a SNK test at *P* < 0.05. Columns labeled with the ^*^ represent significant difference for the comparison between different inoculation treatments at the same level of Cd stress at *P* < 0.05 (SNK test).

DTPA-Cd concentration was significantly improved with increasing level of Cd treatment (Figure 5). However, when subjected to 400 mg kg^−1^ Cd stress, a significant decrease in the DTPA-Cd concentration was detected in the A. aculeatus-inoculated soil compared with non-inoculated soil (Figure 5A).

The pH in the soil slightly increased under the 200 mg kg^−1^ Cd stress compared with the control without Cd stress (Figure 5B). Moreover, there was slight increase in the soil pH for inoculated soil than non-inoculated regime when subjected to three levels of Cd treatment. We found that 400 mg kg^−1^ Cd stress caused a significant decline in the pH of non-inoculated soil. A. aculeatus-inoculated soil had a significant rise in the soil pH compared with non-inoculated soil subjected to 400 mg kg^−1^ Cd exposure, the lowest pH in the soil was detected in the non-inoculated under 400 mg kg^−1^ Cd stress (Figure 5B).

## Discussion

The Bacteria producing IAA, siderophores and ACC deaminase can contribute to plant growth (Glick et al., 1995), which was consistent with our results that A. aculeatus was able to produce IAA, siderophores and ACC deaminase to promote plant growth. In addition, Aspergillus aculeatus has rock phosphate solubilizing ability reported in previous research (Narsian and Patel, 2000). Previous observations showed that the strains PsA4 and Ba32 can protect the plants against the inhibitory effects of chromium, probably through solubilization of phosphate and IAA, siderophores (Rajkumar et al., 2006). These findings support previous study which has demonstrated that root colonizing microorganisms reduce deleterious effects of stresses probably through producing phytohormones (Frankenberger and Arshad, 1995). AMF has been reported to enhance IAA in leaves and roots and ABA in leaves of Lycium barbarum L. grown on saline soils (Liu et al., 2016) and it restored plant biomass which grown on Cu and Zn polluted site although higher Cu and Zn accumulation in roots (Cicatelli et al., 2010). This research suggested that Cd stress caused toxicity to perennial ryegrass. Previous studies have reported that Cd stress has a negative effect on the growth rate of bermudagrass (Xie et al., 2014c). Otherwise, the results not only indicated that Cd stress caused toxicity to perennial ryegrass, but also suggested A. aculeatus had a positive effect on the growth rate of perennial ryegrass. All substrate were sterilized by autoclaving before use to guarantee that it is the A. aculeatus strain that offered this tolerance rather than the total microbial community. Hassan et al. have reported that arbuscular mycorrhizal fungi inoculated on the sunflower could facilitate the growth of plant and alleviate the damage of Cd stress (Hassan et al., 2013). In addition, endophytic bacteria can also promote the growth of rape grown in Pb contaminated soil (Sheng et al., 2008) which supports our result that A. aculeatus-inoculated plants had a significant increase in RGR than non-inoculated regime when exposed to the same levels of Cd treatments (Figure 1).

The turf quality of plants decreased accompanied with the lower RGR, reduced chlorophyll content and the higher the electrolyte leakage under Cd stress. Fortunately, from the results we can deduce that the *A. aculeatus* could help improve the turf quality and chlorophyll content in leaves under Cd stress. It has also been demonstrated that endophytic fungus *Piriformospora indica* colonization increases chlorophyll content, plant biomass and lateral roots density of Arabidopsis under salt stress conditions (Abdelaziz et al., 2017). The degree of cellular damage can be determined according to the cell membrane stability (Saneoka et al., 2004). Hence, the electrolyte leakage which represents the degree of cellular damage was measured to evaluate the toxicity of Cd stress for perennial ryegrass. Remarkably, the results showed that *A. aculeatus-*inoculated plants had a lower electrolyte leakage than Cd only treated regime when exposed to the same Cd treatment, which indicated that *A. aculeatus* can alleviate the toxicity of Cd, such as chlorosis (Das et al., 1997; Zhou and Qiu, 2005). Previous investigation has also demonstrated that Cd stress can lead to chlorosis in plants and even death, while high Cd concentration reduce the chlorophyll content of perennial ryegrass (Milone et al., 2003), which supports the present results. The efficiency of photosynthesis has been reduced for the degradation of chlorophyll (Singh and Dubey, 1995; Luo et al., 2011). It has also documented that Cd could inhibit protochlorophyllide reductase and water-splitting enzyme which presents in the oxidizing site of photosystem II. Eventually, the chlorophyll biosynthesis was affected and the photosynthetic electron transport was also halted (Van and Clijsters, 1990). Chlorophyll *a* fluorescence is a good indicator to describe the degree of impairment which is usually used to study the photosystem of plants exposed to abiotic stress conditions (Chen et al., 2013; Roopin et al., 2013). Here we addressed the question on whether *A. aculeatus* plays any role in perennial ryegrass Cd tolerance by applying chlorophyll *a* fluorescence analysis (OJIP fluorescence transient and JIP-test). The plants treated with Cd showed severe decline of OJIP fluorescence transient than those untreated plants, however, the OJIP fluorescence transient were significantly enhanced in those inoculated plants compared with non-inoculated plants under the corresponding Cd treatments. This discovery implied that *A. aculeatus* had a positive effect on improving the resistance to Cd stress in the perennial ryegrass.

The fluorescence parameters were determined based on the OJIP fluorescence by JIP-test so that the energy flux through PSII could be quantified at the level of reaction center (RC) (Strasser and Strasser, 1995). On the one hand, we discovered that inoculation with *A. aculeatus* remarkably increased the basic parameters including F_M_, F_J_, and F_I_, when the plants were subjected to the same Cd treatments. On the other hand, the M_0_, V_I_, and V_J_ values were all decreased notably in *A. aculeatus*-inoculated plants vs. non-inoculated. The significant difference in these parameters implied that *A. aculeatus* was actively responsible for Cd resistance of perennial ryegrass and can alleviate Cd toxicity on PSII system. The performance index PI_total_ is an important sensitive parameter to assess photochemical activities of the plant under the stress situation. It incorporates several independent parameters such as RC/ABS, ϕP_0_ and ΨE_0_ calculated from fluorescence transient OJIP (Clark et al., 2000; Yusuf, 2010). In our study, PI_total_ and PI_ABS_ were both greatly enhanced after inoculation with *A. aculeatus* under the Cd treatments, which indicated the protective role of *A. aculeatus* in Cd resistance. Furthermore, ϕP_0_, ϕR_0_, and ϕE_0_ which reflected Quantum yields and efficiencies were strongly improved by *A. aculeatus* when exposed to Cd stress. These findings suggested that *A. aculeatus* could affect the quantum yield of donor and acceptor located at different sides of PSII. In addition, grasses inoculated with *A. aculeatus* had a less decrease in ABS/RC and TR_0_/RC in contrast with those that were non-inoculated. These results further demonstrated that *A. aculeatus* could alleviate the damage in PSII RC caused by Cd toxicity.

*A. aculeatus* not only improved the efficiency of photosynthesis but also affected the content of TOC and TN in plants. In this study, the remarkable decline in shoot TN and root TOC under Cd stress implied that Cd stress might inhibit the transportation of C from leaves to roots and the N from root to shoot. Previous findings showed that Cd treatment caused an accumulation of NO_3_^−^ in the root and changed the translocation of NO_3_^−^ to the shoot (Hernandez et al., 1996). However, the greater accumulation of N in root resulted in the lower C/N ratio under the Cd treatments which indicated that Cd stress disturbed the balance between carbon metabolism and nitrogen metabolism in plants. Previous investigation had reported that the C/N in seagrass was decreased after exposure to diuron (a kind of herbicide) for 11 weeks (Negri et al., 2015). We hypothesized that Cd stress reduced the C/N ratio by declining photosynthetic C assimilation. Conversely, C/N ratios were enhanced in the shoots in the presence of Cd, which might be attributed to the lower accumulation of N. It has been demonstrated that Cd stress can inhibit NO_3_^−^ assimilation in the shoot by declining relevant nitrate reductase (NR) activity (Hernandez et al., 1996). In addition, the plants inoculated with *A. aculeatus* significantly enhanced the C/N ratios by increasing the accumulation of N in shoot. This result further confirmed that *A. aculeatus* could improve the resistance to Cd stress in perennial ryegrass and relieve the damage to plant metabolism caused by Cd stress.

Additionally, the C/N ratios, the ionic homeostasis can also be regulated by *A. aculeatus*. It has been demonstrated that Cd can alter the concentration of mineral nutrition such as Mn in pea seedlings (Hernandez et al., 1996). It is more likely that *P. indica* colonization regulated ion homeostasis of Na^+^/K^+^ to promote Arabidopsis growth under salt stress (Abdelaziz et al., 2017). Different ions in different tissue of plant showed different ionic response to Cd stress. In this study, Mg concentration decreased in root but increased in shoot with the increasing Cd stress, which indicated the Cd stress might facilitate the transport of Mg from root to shoot. On the other hand, the Ca, P, and Mn concentrations in the root were severely affected among several ionic nutrients studied (i.e. Ca, Cu, Mg, Mn, or P), which were significantly increased by the inoculation of *A. aculeatus* compared with non-inoculated plants. Previous studies have reported that the hyphae of mycorrhizal fungus could efficiently absorb P (Li et al., 1991; Rufyikiri et al., 2004). Arbuscular mycorrhizal (AM) fungus and the root of host plants formed a symbiosis, in which nutrients could be transported in both directions (Smith and Read, 1997). Mycorrhizal inoculation improved cork oak forest resistance capacity to deal with coming climate change by forming symbiotic relationship and improve root capacity to take up nutrients (Sebastiana et al., 2017). Hence, we assumed that these significant differences were attributed to the interaction mechanism between the fungus and the root of perennial ryegrass which can help alleviate the Cd toxicity. A further investigation is required to clarify the integrated mechanism between fungus and perennial ryegrass under the Cd stress.

Generally, this study deduced that inoculation with *A. aculeatus* significantly decreased the available Cd in the soil. Previously, DTPA, which is a kind of chelate, was commonly used for extracting metals from soils (Misra et al., 1990; Erickson et al., 1991; Hughes and Noble, 1991) especially for heavy metals such as Cd, Zn, Pb (Ma et al., 1997). Recently, DTPA-Cd, which is recognized as a high-bioavailability chemical form of Cd has become a more available indicator to estimate the amount of Cd absorbed by plants and their toxicity effects for plants. Previous reports have confirmed that DTPA-extractable concentrations are more available to plants due to their higher mobility (Lindsay and Norvell, 1978; Petruzzelli, 1989; Ma et al., 1997; Zufiaurre et al., 1998). The DTPA-Cd concentration in the soil was significantly enhanced with the increasing level of Cd treatment. Meanwhile, *A. aculeatus* resulted in a remarkable decrease of DTPA-Cd when exposed to 400 mg kg^−1^ Cd stress. Interestingly, the highest concentration of DTPA-Cd was found in the soils which were non-inoculated and with lower soil pH. This was more likely to have resulted from the low soil pH values. Similar observations have been made in previous study using carambola trees (Li et al., 2006). The pH and organic matter were confirmed to be two critical factors affecting the heavy metal from soil absorbed by plants (Mclaughlin et al., 1999; Evangelou et al., 2004). Moreover, it has been demonstrated that soil pH is closely related to heavy metals bioavailability (Li et al., 2006). In addition, Cd existed mainly as free ions forms in the acidic pH soil solutions (Ge et al., 2000) hence we inferred that Cd has a higher bioavailability in the relative lower pH. However, the present results showed that soil pH was higher in the *A. aculeatus-*inoculated soil compared with uninfected soil. Therefore, we presumed that the *A. aculeatus* could help to reduce Cd availability in the soil by improving the soil pH.

Furthermore, we found that the Cd concentration in shoots were lower in *A. aculeatus-*inoculated plants than Cd only treated regime when objected to the same Cd treatments due to the lower available Cd in the soil caused by *A. aculeatus*. Otherwise, a previous investigation has also reported that *A. aculeatus-*inoculated bermudagrass have a lower Cd concentration in leaves (Xie et al., 2014b) which is consistent with our results. The present study suggested that inoculation with *A. aculeatus* inhibited the Cd transportation to shoots and similar results have been reported in the sunflowers inoculated with *F. mosseae* strain (Hassan et al., 2013). Moreover, another previous study indicated that the Cd-tolerant genotype WB242 of bermudagrass accumulated less Cd in the shoot when subjected to Cd stress (Xie et al., 2014a), which also supports our results. The *A. aculeatus* decreased available Cd in the soil and accumulated much Cd in their mycelia which led to the lower Cd level in plant so that it can filter out Cd and prevent absorption by roots.

## Conclusions

**Figure 6:**
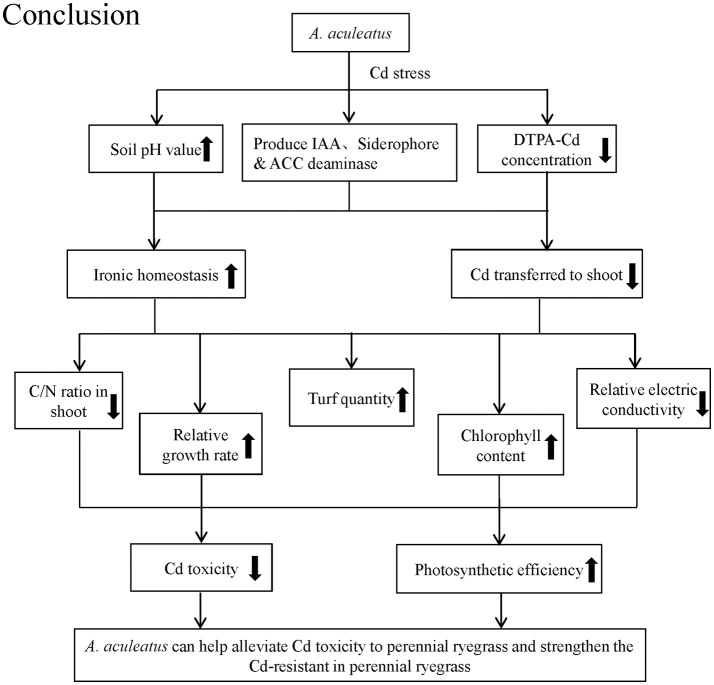
The conclusions about how *A. aculeatus* affected perennial ryegrass when exposed to different levels of Cd concentrations.

The *A. aculeatus* strains colonizing the roots outer epidermal interiors might promote the plant growth and alleviate the toxic effects of high concentrations of Cd by secreting IAA, siderophore or ACC deaminase. In summary, *A. aculeatus* could alleviate Cd toxicity in their host plants and strengthen the Cd-resistant in perennial ryegrass by improving soil pH to reduce available Cd in soil, inhibiting the uptake of Cd by plants. Through these mechanisms, *A. aculeatus* facilitated the growth rate of perennial ryegrass under Cd stress, remarkably improved turf quality, chlorophyll content and photosynthetic efficiency and significantly decreased the electrolyte leakage. Moreover, the nitrogen and carbon metabolism and ionic homeostasis were all regulated by *A. aculeatus* to defense against Cd damage (Figure 6). All these evidences had confirmed that *A. aculeatus* is a potential candidate to be applied into the phytostabilization of Cd contaminated soil. However, there might be other unknown mechanisms underlying this phenomenon and thus further research on relationship between the plant and this fungus is essential.

## Author contributions

JF and YX designed the experiments; SH and XL performed the experiments, analyzed the data and wrote the manuscript; EA, YX, and JF revised the manuscript. All authors approved the final manuscript and declare no competing financial interests.

### Conflict of interest statement

The authors declare that the research was conducted in the absence of any commercial or financial relationships that could be construed as a potential conflict of interest.
